# Adverse cardiovascular, limb, and renal outcomes in patients with diabetes after peripheral artery disease revascularization treated with sodium glucose cotransporter 2 inhibitors versus dipeptidyl peptidase-4 inhibitors

**DOI:** 10.1186/s13098-023-00982-6

**Published:** 2023-01-18

**Authors:** Hsin-Fu Lee, Chi Chuang, Pei-Ru Li, Yung-Hsin Yeh, Yi-Hsin Chan, Lai-Chu See

**Affiliations:** 1 Division of Cardiology, Department of Internal Medicine, New Taipei City Municipal Tucheng Hospital, Taoyuan City, Taiwan; 2 The Cardiovascular Department, Chang Gung Memorial Hospital, Linkou, Taoyuan City, 33305 Taiwan; 3 College of Medicine, Chang Gung University, Taoyuan City, 33302 Taiwan; 4 Graduate Institute of Clinical Medical Sciences, College of Medicine, Chang Gung University, Taoyuan City, Taiwan; 5 Department of Public Health, College of Medicine, Chang Gung University, Taoyuan City, 33302 Taiwan; 6 Microscopy Core Laboratory, Guishan Dist, Chang Gung Memorial Hospital, No.259, Wenhua 1St Rd, Taoyuan City, 33302 Taiwan; 7 Biostatistics Core Laboratory, Molecular Medicine Research Center, Chang Gung University, Taoyuan City, 33302 Taiwan; 8 Division of Rheumatology, Allergy and Immunology, Department of Internal Medicine, Chang Gung Memorial Hospital, Linkou, Taoyuan City, 33305 Taiwan

**Keywords:** Sodium glucose co-transporter 2 inhibitors, Peripheral artery disease, Revascularization, Diabetes, Dipeptidyl peptidase-4 inhibitors, Amputation, Cardiovascular

## Abstract

**Aims:**

The effectiveness and limb safety of sodium glucose co-transporter 2 inhibitors (SGLT2i) for patients with type-2 diabetes (T2D) who have received peripheral artery disease (PAD) revascularization are unknown.

**Methods and results:**

In this nationwide retrospective cohort study, we identified a total of 2,455 and 8,695 patients with T2D who had undergone PAD revascularization and received first prescriptions for SGLT2i and dipeptidyl peptidase-4 inhibitors (DPP4i), respectively, between May 1, 2016, and December 31, 2019. We used 1:1 propensity score matching (PSM) to balance covariates between the two study groups. Patients were followed up from the drug index date until the occurrence of specified outcomes, death, discontinuation of the index drug, or the end of the study period, whichever occurred first. After PSM, we observed that compared with DPP4i, SGLT2i were associated with comparable risks of ischemic stroke, acute myocardial infarction, and heart failure hospitalization but were associated with a lower risk of cardiac death (hazard ratio [HR]: 0.60; 95% confidence interval [CI]: 0.40–0.90]; *p* = 0.0126). Regarding major limb outcomes, SGLT2i were associated with comparable risks of repeated revascularization and lower limb amputation compared with DPP4i. SGLT2i were associated with a lower risk of composite renal outcomes (HR: 0.40; 95% CI: 0.27–0.59; *p* < 0.0001) compared with DPP4i.

**Conclusion:**

In a real-world study of patients with T2D who had undergone PAD revascularization, SGLT2i were associated with lower risks of cardiac death and composite renal outcomes but not associated with increased risks of adverse limb events compared with DPP4i.

**Supplementary Information:**

The online version contains supplementary material available at 10.1186/s13098-023-00982-6.

## Introduction

Type-2 diabetes (T2D) affects people worldwide and increases the risk of adverse cardiovascular events and can even result in cardiac death [1]. Patients with T2D and peripheral artery disease (PAD) are at a higher risk of cardiovascular events and lower limb amputation. Therefore, patients who still exhibit claudication or critical limb ischemia despite receiving optimal medical therapy are referred for bypass surgery or endovascular therapy to aid in revascularization [2]. Since 2008, the novel antihyperglycemic agents: sodium–glucose cotransporter-2 inhibitors (SGLT2i) and dipeptidyl peptidase-4 inhibitors (DPP4i) have been subjected to several trials to investigate their cardiovascular outcomes. These trials have demonstrated that SGLT2i are beneficial for reducing the risks of heart failure and other major adverse cardiovascular events [3–5] but that DPP4i exhibit neutral effects in terms of cardiovascular composite outcomes for patients with T2D [6–9]. During the study period, SGLT2i and DPP4i were widely prescribed as second-line agents for the management of hyperglycemia in patients with T2D directed by the guideline, and SGLT2i were advocated for patients at a high risk of cardiovascular events [10]. However, canagliflozin was reported to be associated with a higher rate of amputations compared with a placebo in the Canagliflozin Cardiovascular Assessment Study (CANVAS) trial [3]. These findings have raised concerns about the general safety of SGLT2i for patients with T2D and concomitant PAD, who are inherently at an increased risk of limb amputation [11]. In addition, perioperative acute kidney injury developed in patients with PAD who underwent endovascular or surgical revascularization was associated with high risks of adverse renal outcomes and all-cause mortality [12]. Renal dysfunction was an independent predictor for adverse cardiovascular and limb events in patients after PAD revascularization, that demonstrating the importance of renal protection in this specific group [13]. Furthermore, limited clinical trials or real-world data are available regarding the cardiovascular, limb, or renal outcomes of these antihyperglycemic agents when administered to patients with T2D after PAD revascularization. Accordingly, we conducted this population-based nationwide cohort study with the aim of comparing the cardiovascular, limb, and renal outcomes of SGLT2i and DPP4i in patients with T2D after PAD revascularization treatment.

## Methods

### Study population and cohort

**Fig. 1:**
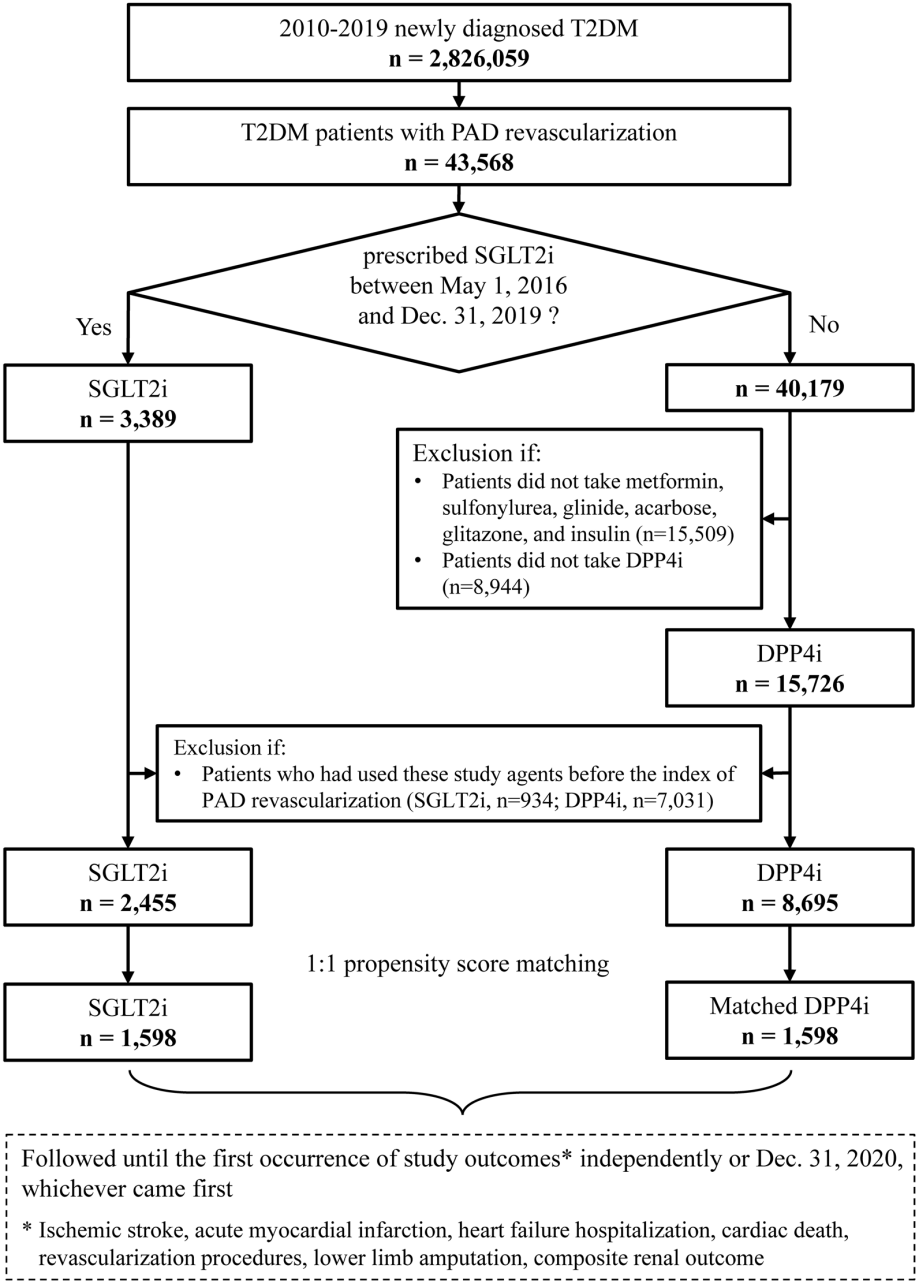
Enrollment of patients with T2D who were treated with SGLT2i or DPP4i after PAD revascularization. *DPP4i* dipeptidyl peptidase-4 inhibitors, *PAD* peripheral artery disease, *SGLT2i* sodium–glucose cotransporter-2 inhibitors, *T2D* type 2 diabetes

This nationwide retrospective cohort study enrolled patients from the Taiwan National Health Insurance Research Database (NHIRD), which contains health-care information for more than 23 million (> 99%) residents of Taiwan [14]. From a cohort of 2,826,059 patients with T2D—diagnosed using *International Classification of Diseases, Ninth Revision, Clinical Modification* (*ICD-9-CM*) code 250 (between 2010 and 2015) or *International Classification of Diseases, Tenth Revision, Clinical Modification* (*ICD-10-CM*) codes E11 and E13 (between 2016 and 2019)—we identified 43,568 patients who had undergone PAD revascularization. Of the identified patients, 17,975 had been treated with SGLT2i (*n* = 3,389) or DPP4i (*n* = 15,726). After excluding patients who had used these study agents before the index date of PAD revascularization, we identified a total of 2,455 and 8,695 patients who had received first prescriptions for SGLT2i (empagliflozin, dapagliflozin, or canagliflozin) and DPP4i (saxagliptin, vildagliptin, sitagliptin, linagliptin, or alogliptin) during the study period, respectively. Notably, according to Taiwan’s National Health Insurance regulations, patients with T2D cannot use SGLT2i and DPP4i simultaneously. The index date for each study group was defined as the date of the first prescription for SGLT2i or DPP4i after PAD revascularization. The follow-up period was defined as the time from the index date to the independent occurrence of any study outcome, discontinuation of the index drug, or the end of the study period (December 31, 2020), whichever occurred first. The patient enrollment flowchart is illustrated in **Fig. ****1**. The Institutional Review Board of Chang Gung Medical Foundation approved this study **(**201801427B0**)**. Informed consent was waived because the original identification number of each patient in the NHIRD had been encrypted and deidentified to protect their privacy.

### Covariates and study outcomes

All baseline covariates were obtained from the NHIRD through the analysis of claims records associated with diagnoses, procedures, or medication codes predating the index date. Prescription medications were confined to medications used at least once within 3 months before the index date. The study outcomes were as follows: (1) cardiovascular outcomes (ischemic stroke [IS], acute myocardial infarction [AMI], heart failure hospitalization [HFH], or cardiac death), (2) limb outcomes (repeated revascularization procedures such as endovascular therapy or bypass surgery or lower limb amputation), and (3) composite renal outcomes (dialysis, renal transplantation, death from renal causes, or hospitalization for renal events). The diagnostic codes used in the NHIRD to identify these outcomes were based on *ICD-9-CM* (through 2015) and *ICD-10-CM* (after January 1, 2016) and are summarized in Additional file 1: Tables S1 and S2, respectively**.**

### Statistical analysis

We used propensity score matching (PSM) to balance the baseline covariates between the two study groups (i.e., SGLT2i and DPP4i groups) [15]. We calculated propensity scores for all covariates in Table 1 by using a generalized boosted model (GBM). The GBM involves an iterative process with multiple regression trees to capture complex and nonlinear relationships between treatment assignment and pretreatment covariates without overfitting the data, thus leading to the best balance across study groups [16]. We performed 1:1 PSM between the SGLT2i and DPP4i groups without replacement and with nearest-neighbor matching within a caliper width (8-to-1 digit matching) [17]. Furthermore, we derived absolute standardized mean difference (ASMD) values rather than performing statistical testing to assess the balance of potential confounders at baseline (index date) between the two groups; this is because balance is a property of the sample and not the underlying population. An ASMD value of ≤ 0.1 was considered to indicate a nonsignificant difference in potential confounders between the two study groups [18]. We derived the incidence of outcomes as the total number of study outcomes during the follow-up period divided by person-years at risk. The risk of study outcomes throughout the follow-up period was obtained using the Kaplan–Meier method and log-rank test. A Cox proportional hazards model was made to derive the hazard ratio (HR) and the corresponding with 95% confidence interval (CI) for SGLT2i (experimental group) versus DPP4i (reference). Because we used PSM to balance the baseline characteristics of the study groups, we included only the study groups in the Cox model [13]. Statistical significance was defined as a *p* value of < 0.05. All statistical analyses were performed using SAS 9.4 (SAS Institute, Cary, NC, USA).

**Table 1:** Baseline characteristics of patients with T2D who had undergone PAD revascularization taking SGLT2i or DPP4i before and after propensity score matching

	Before PSM					After PSM				
	SGLT2i		DPP4i		ASMD	SGLT2i		DPP4i		ASMD
	(n = 2,455)		(n = 8,695)			(n = 1,598)		(n = 1,598)		
Age (mean)	67.3 ± 11.0	70.8 ± 11.2	0.3166	67.5 ± 11.3	68.5 ± 10.9	0.0534				
< 65	961	39.14%	2477	28.49%	0.3217	550	34.42%	541	33.85%	0.0438
65–74	815	33.20%	2660	30.59%		526	32.92%	535	33.48%	
75–84	556	22.65%	2594	29.83%		417	26.10%	430	26.91%	
> 85	123	5.01%	964	11.09%		105	6.57%	92	5.76%	
Male	1717	69.94%	5120	58.88%	0.2324	1073	67.15%	1069	66.90%	0.0053
Chronic liver disease	326	13.28%	1177	13.54%	0.0076	207	12.95%	216	13.52%	0.0166
Chronic kidney disease	863	35.15%	5364	61.69%	0.5507	650	40.68%	600	37.55%	0.0641
Congestive heart failure	179	7.29%	989	11.37%	0.1407	110	6.88%	102	6.38%	0.0201
Hypertension	2184	88.96%	8065	92.75%	0.1319	1422	88.99%	1426	89.24%	0.008
Dyslipidemia	2130	86.76%	6679	76.81%	0.2599	1336	83.60%	1358	84.98%	0.0378
Ischemic stroke	605	24.64%	2047	23.54%	0.0258	400	25.03%	409	25.59%	0.0129
Ischemic heart disease	644	26.23%	1599	18.39%	0.1892	319	19.96%	334	20.90%	0.0233
Gout	497	20.24%	1907	21.93%	0.0414	338	21.15%	332	20.78%	0.0092
Malignancy	248	10.10%	1084	12.47%	0.0748	173	10.83%	166	10.39%	0.0142
PCI	817	33.28%	2382	27.40%	0.1282	438	27.41%	429	26.85%	0.0127
CABG	134	5.46%	393	4.52%	0.0431	70	4.38%	66	4.13%	0.0124
Diabetic medications										
Use of metformin	1217	49.57%	2287	26.30%	0.4939	801	50.13%	862	53.94%	0.0764
Use of sulfonyurea	1304	53.12%	3274	37.65%	0.3143	754	47.18%	787	49.25%	0.0413
Use of glinides	218	8.88%	1971	22.67%	0.3852	161	10.08%	152	9.51%	0.0189
Use of acarbose	342	13.93%	898	10.33%	0.1105	178	11.14%	184	11.51%	0.0118
Use of glitazones	374	15.23%	488	5.61%	0.3188	158	9.89%	169	10.58%	0.0227
Use of insulin	833	33.93%	3985	45.83%	0.2448	602	37.67%	576	36.05%	0.0337
CV medications										
Use of APTs	1834	74.70%	6040	69.47%	0.117	1157	72.40%	1177	73.65%	0.0282
Use of ACEI/ARB	1665	67.82%	4925	56.64%	0.2321	1053	65.89%	1080	67.58%	0.0359
Use of amiodarone	124	5.05%	640	7.36%	0.0958	75	4.69%	82	5.13%	0.0203
Use of dronedarone	4	0.16%	19	0.22%	0.0127	3	0.19%	2	0.13%	0.0158
Use of beta-blocker	1318	53.69%	4102	47.18%	0.1305	804	50.31%	822	51.44%	0.0225
Use verapamil/diltiazem	208	8.47%	765	8.80%	0.0116	132	8.26%	130	8.14%	0.0046
Use of digoxin	91	3.71%	324	3.73%	0.001	56	3.50%	53	3.32%	0.0103
Use of statin	1924	78.37%	4590	52.79%	0.559	1138	71.21%	1202	75.22%	0.0905
Use of DOACs	253	10.31%	450	5.18%	0.1928	144	9.01%	156	9.76%	0.0257
Use of warfarin	103	4.20%	532	6.12%	0.087	66	4.13%	57	3.57%	0.0293
Use of loop diuretics	501	20.41%	1869	21.50%	0.0267	308	19.27%	318	19.90%	0.0158
Use of MRA	304	12.38%	685	7.88%	0.1497	163	10.20%	170	10.64%	0.0143
Use of ARNI	70	2.85%	51	0.59%	0.1749	22	1.38%	26	1.63%	0.0206

*ACEI* angiotensin-converting-enzyme inhibitor, *APT* antiplatelet agent, *ARB* angiotensin II receptor antagonist, *ARNI* angiotensin receptor-neprilysin inhibitor, *ASMD* absolute standardized mean difference, *CABG* coronary artery bypass graft, *CV* cardiovascular, *DPP4i* dipeptidyl peptidase-4 inhibitors, *DOAC* direct oral anticoagulant, *MRA* mineralocorticoid receptor antagonist, *PAD* peripheral artery disease, *PCI* percutaneous coronary intervention, *PPI* proton pump inhibitor, *SGLT2i* sodium glucose co-transporter-2 inhibitors, *T2D* type-2 diabetes

#### Results

### Baseline characteristics of SGLT2i and DPP4i groups

Among patients with T2D who had undergone PAD revascularization, 2,455 and 8,695 had received first prescriptions for SGLT2i and DPP4i, respectively, between May 1, 2016, and December 31, 2019. The SGLT2i agents were dapagliflozin, empagliflozin, and canagliflozin and were prescribed to 997 (40.61%), 1305 (53.16%), and 153 (6.23%) patients, respectively. The DPP4i agents were sitagliptin, vildagliptin, linagliptin, saxagliptin, and alogliptin and were prescribed to 1,875 (21.56%), 1,780 (20.47%), 4,436 (51.02%), 562 (6.46%), and 42 (0.48%) patients, respectively. Before PSM, we observed that compared with the DPP4i group, the SGLT2i group was younger; had a male predominance; had a lower prevalence of chronic kidney disease (CKD), hypertension, and malignancy; had a higher prevalence of dyslipidemia, ischemic heart disease, and history of percutaneous coronary intervention; and had a higher rate of prescriptions for metformin, sulfonylurea, acarbose, glitazones, antiplatelet agents, angiotensin-converting enzyme inhibitors/angiotensin II receptor antagonists (ACEIs/ARBs), beta blockers, statins, direct oral anticoagulants, mineralocorticoid receptor antagonists, and angiotensin receptor–neprilysin inhibitors (ASMD > 0.1). After PSM, the two study groups were well balanced in all baseline characteristics (ASMD < 0.1; **Table ****1**).

### Main analysis of SGLT2i versus DPP4i

**Fig. 2:**
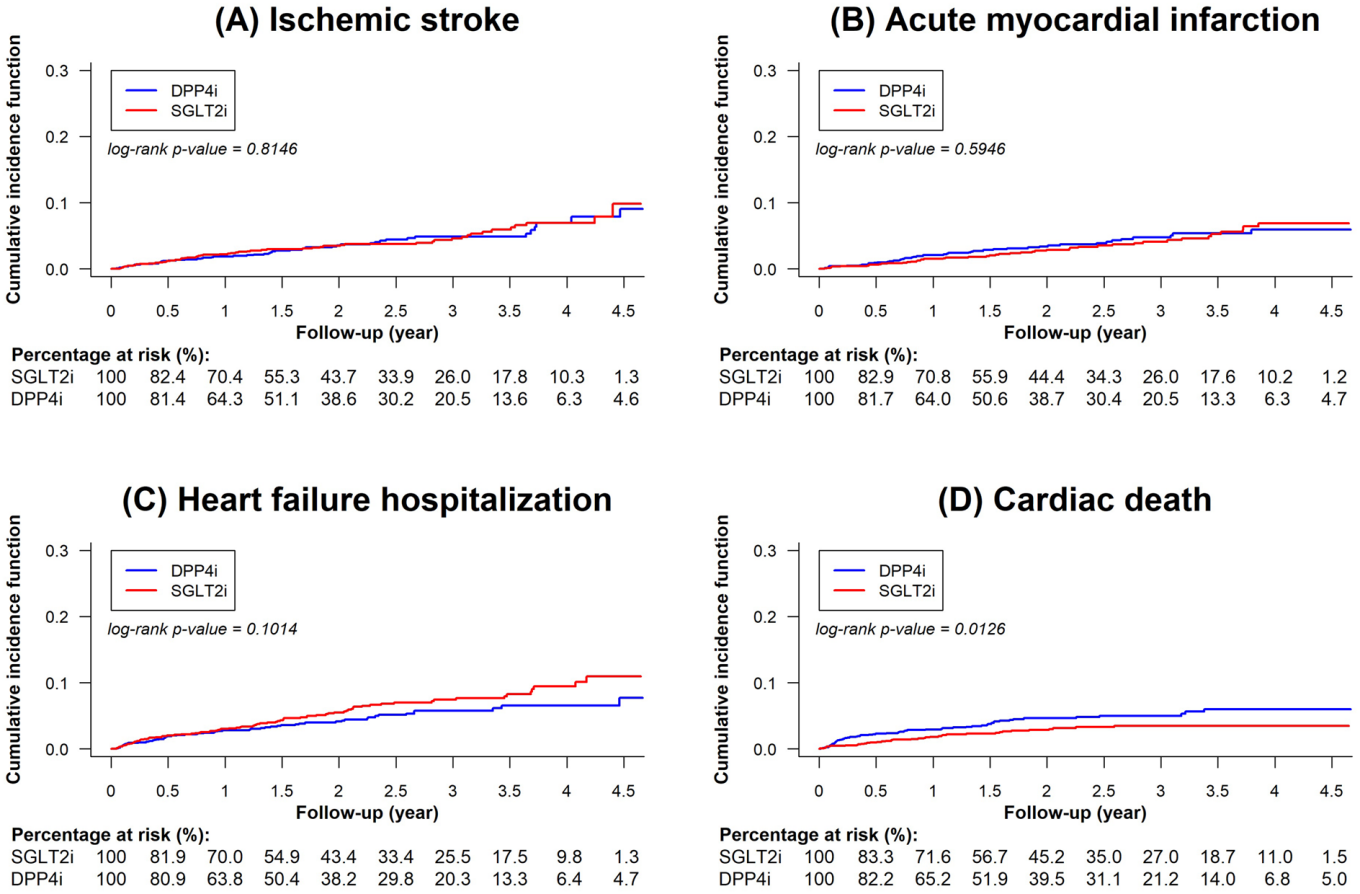
Cumulative incidence curves of cardiovascular outcomes for SGLT2i versus DPP4i (after PSM) among patients with T2D who had undergone PAD revascularization. Cumulative incidence curves of specified outcomes of SGLT2i versus DPP4i (after PSM) among patients with T2D who had undergone PAD revascularization: **(A)** IS, **(B)** AMI, **(C)** HFH, and **(D)** cardiac death are presented. SGLT2i were associated with comparable cumulative risks of IS, AMI, and HFH and a lower cumulative risk of cardiac death compared with DPP4i. *AMI* acute myocardial infarction, *HFH* heart failure hospitalization, *IS* ischemic stroke, *PSM* propensity score matching. Other abbreviations are the same as those in Fig. 1

**Fig. 3:**
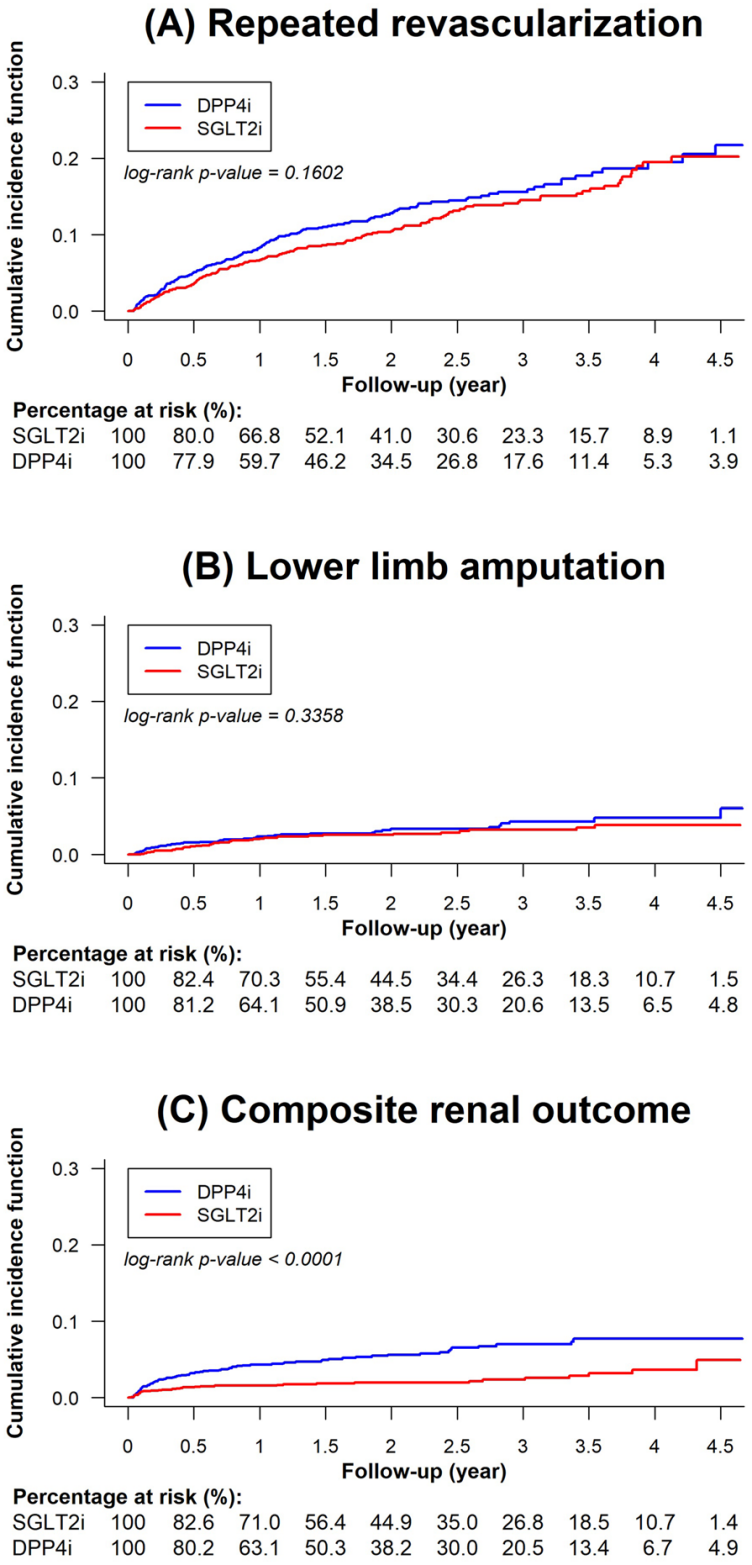
Cumulative incidence curves of limb outcomes and composite renal outcomes of SGLT2i versus DPP4i (after PSM) among patients with T2D who had undergone PAD revascularization. Cumulative incidence curves of specified outcomes of SGLT2i versus DPP4i (after PSM) among patients with T2D who had undergone PAD revascularization: **a** repeated revascularization, **b **lower limb amputation, **c** and composite renal outcomes are presented. Compared with DPP4i, SGLT2i were associated with comparable cumulative risks of repeated revascularization and lower limb amputation but a lower cumulative risk of composite renal outcomes. Other abbreviations are the same as those in Figs. 1 and 2

**Fig. 4:**
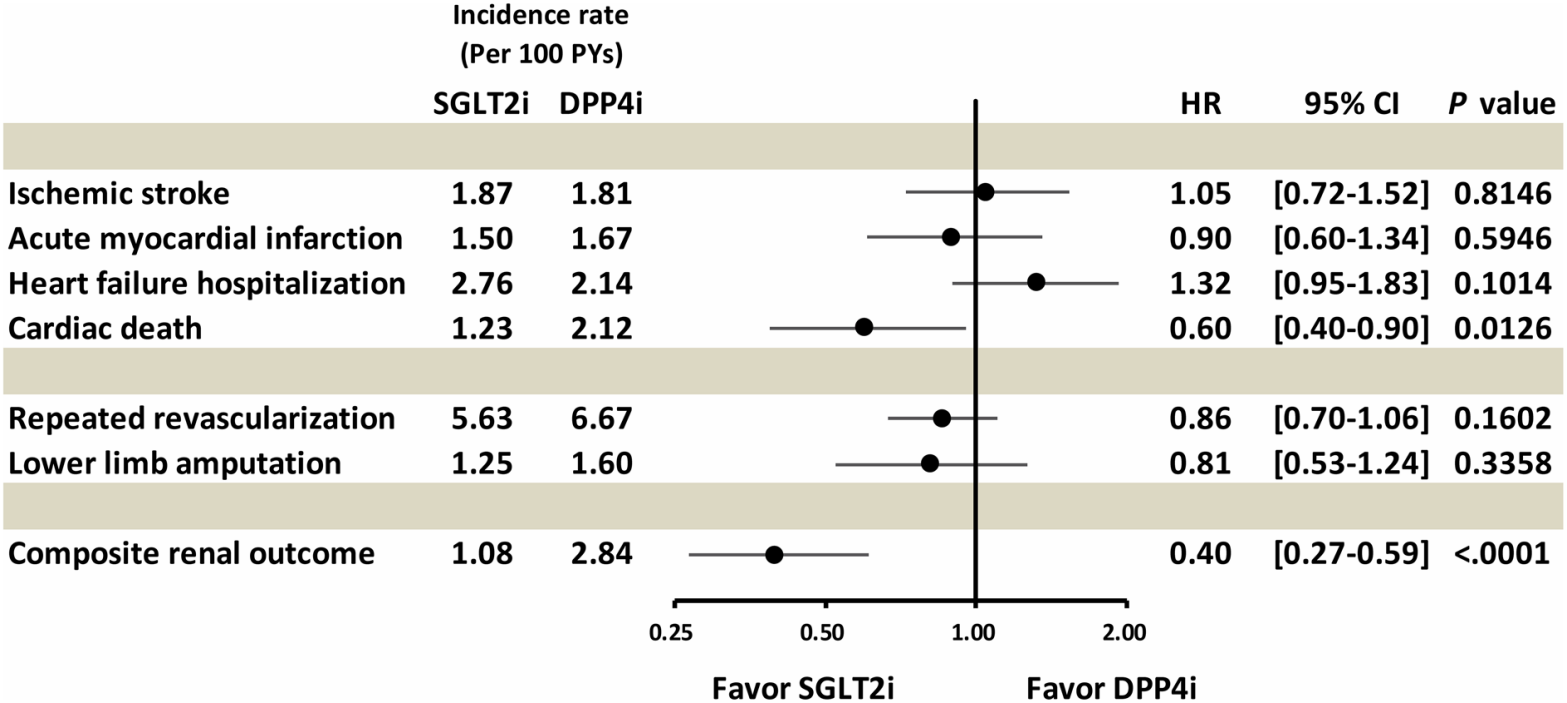
Forest plot of the hazard ratios of clinical outcomes of SGLT2i versus DPP4i (after PSM) among patients with T2D who had undergone PAD revascularization. Compared with DPP4i, SGLT2i were associated with comparable risks of IS, AMI, HFH, repeated revascularization, and lower limb amputation but were associated with lower risks of cardiac death and composite renal outcomes. *HR* hazard ratio. Other abbreviations are the same as those in Figs. 1 and 2

After PSM, we noted that the SGLT2i and DPP4i groups had comparable incidence rates of IS (1.87 vs. 1.81 per 100 person-years, *p* = 0.8146), AMI (1.50 vs. 1.67 per 100 person-years, *p* = 0.5946), and HFH (2.76 vs. 2.14 per 100 person-years, *p* = 0.1014). The SGLT2i group was significantly associated with a lower incidence rate of cardiac death compared with the DPP4i group (1.23 vs. 2.12 per 100 person-years; HR: 0.60; 95% CI: 0.40–0.90; *p* = 0.0126; Table 2, Figs. 2 and 4). Regarding limb outcomes, after PSM, we observed that the SGLT2i and DPP4i groups had comparable cumulative risks of repeated revascularization (5.63 vs. 6.67 per 100 person-years, *p* = 0.1602) and lower limb amputation (1.25 vs. 1.60 per 100 person-years, *p* = 0.3358). Moreover, after PSM, we determined that the SGLT2i group was associated with a lower cumulative risk of composite renal outcomes compared with the DPP4i group (1.08 vs. 2.84 per 100 person-years, *p* < 0.0001; Table 2, and Figs. 3 and 4).

**Table 2:** Number of events, event rates, and hazard ratios (HR) for SGLT2i versus DPP4i among patients with T2D who had undergone PAD revascularization after propensity score matching

	SGLT2i		DPP4i		Cox model	
	(n = 1,598)		(n = 1,598)			
	Number	Incidence rate per 100 person-year	Number	Incidence rate per 100 person-year	HR (95% CI)	P value
Cardiovascular outcomes						
Ischemic stroke	58	1.87	52	1.81	1.05 (0.72–1.52)	0.8146
Acute myocardial infarction	47	1.50	48	1.67	0.81 (0.52–1.26)	0.5946
Heart failure hospitalization	85	2.76	61	2.14	1.39 (0.97–1.99)	0.1014
Cardiac death	39	1.23	62	2.12	0.60 (0.40–0.90)	0.0126
Limb outcomes						
Repeated revascularization	165	5.63	177	6.67	0.86 (0.70–1.06)	0.1602
Lower limb amputation	39	1.25	46	1.60	0.81 (0.53–1.24)	0.3358
Composite renal outcomes	34	1.08	81	2.84	0.40 (0.27–0.59)	< 0.0001

*CI* confidence interval, *DPP4i* dipeptidyl peptidase-4 inhibitors, *HR* hazard ratio, *PAD* peripheral artery disease, *SGLT2i* sodium glucose co-transporter-2 inhibitors, *T2D* type-2 diabetes

### Subgroup analysis of SGLT2is versus DPP4is

Overall, the subgroup analysis revealed consistent results for most outcomes of SGLT2i versus DPP4i among patients aged ≥ 75 years, patients with CKD, female patients, and patients who used statins; these results were consistent with the main analysis results (*p* for interaction > 0.05; Additional file 1: Figures SI to SVII). The subgroup analysis indicated that SGLT2is reduced the risk of AMI, cardiac death, and composite renal outcomes in patients without concomitant metformin therapy but not in those with metformin therapy (*p* < 0.05; Additional file 1: Figures SII, IV, and VII). The subgroup analysis of patients with concomitant CKD revealed a lower risk of repeated revascularization for SGLT2i versus DPP4i (*p* < 0.05; Additional file 1: Figure SV).

## Discussion

To the best of our knowledge, this is the first nationwide cohort study to compare the cardiovascular and limb outcomes of SGLT2i and DPP4i administered to patients with T2D who had undergone PAD revascularization. The main findings of this study are that compared with DPP4i, SGLT2i were associated with comparable risks of IS, AMI, and HFH but were not associated with a higher risk of repeated revascularization or lower limb amputation. However, SGLT2i were associated with lower risks of cardiac death and composite renal outcomes compared with DPP4i. These observations persisted among several major subgroups.

Although SGLT2i and DPP4i are both commonly prescribed as second-line agents for glycemic control in patients with T2D (with metformin as the first-line agent), our study revealed differences in preferences in the prescription of these agents in our cohort. Specifically, we observed that the SGLT2i group exhibited higher prevalence of ischemic heart disease, hypertension, dyslipidemia, and history of percutaneous coronary intervention but a lower prevalence of CKD. A likely explanation is that SGLT2i were prescribed to patients at a high risk of cardiovascular events because these agents are beneficial for reducing the risks of cardiovascular events; they were not prescribed to patients with advanced kidney disease because they engender glycuresis through the kidneys [10]. We observed that SGLT2is reduced the risk of AMI, cardiac death, and composite renal outcomes in patients without concomitant metformin therapy but not in those with metformin therapy in subgroup analysis. Although metformin had been reported to have cardioprotective effects, it may increase the risk of lactate acidosis in patients with CKD and the risk of acute kidney injury during revascularization with contrast medium administration that contribute to the worse outcomes in our study population [19].

Patients with T2D and concomitant PAD have a higher risk of mortality and amputation than do those with T2D or PAD alone [11, 20]. PAD revascularization was suggested for patients who exhibited claudication or critical limb ischemia despite optimal medical therapy [2]. Notably, patients with T2D who have received PAD revascularization are generally considered to have advanced-stage PAD, which is associated with poor cardiovascular and limb outcomes [21–23]. Previous studies have revealed that intensive glycemic control may improve outcomes in this high-risk group [24–26], but few studies have investigated the outcomes of different antihyperglycemic agents in patients with T2D who have undergone PAD revascularization. Several large-scale clinical trials have indicated that DPP4i had a neutral effect on cardiovascular composite outcomes in patients with T2D [6–9]; nevertheless, these trials did not explore the outcome of adverse lower limb events. Other landmark clinical trials have demonstrated that SGLT2i are beneficial in reducing the risks of HFH and other major adverse cardiovascular events [3–5]. However, the CANVAS trial reported a higher rate of amputations in the canagliflozin (SGLT2i) group compared with the placebo group (0.63 vs. 0.34 per 100 person-year, *p* < 0.001) [3]. This adverse reaction can be attributed to the diuretic effect of SGLT2i, which caused volume depletion, leading to inadequate circulation in the distal peripheral vasculature [3, 27]. In addition, previous cohort studies including patients with T2D have revealed that SGLT2i were associated with an increased risk of amputation compared with other antihyperglycemic agents [28–30]. By contrast, a meta-analysis revealed no significant association between SGLT2is and an increased risk of amputation [31]. A large-scale observational study of patients with T2D and concomitant PAD reported that SGLT2i were associated with lower risks of adverse lower limb events compared with DPP4i [32]. Accordingly, evidence concerning the association between SGLT2i use and the risk of lower limb amputation is inconclusive. Our data reveal that the SGLT2i and DPP4i groups had similar incidence rates of repeated revascularization (5.63 and 6.67 per 100 person-years, respectively) and lower limb amputation (1.25 and 1.60 per 100 person-years, respectively), to the findings of a relevant study that included patients who had received PAD revascularization [23]. These findings in such a high-risk population support that SGLT2i are safe glycemic control agents for patients with T2D and concomitant PAD, even after revascularization.

A previous meta-analysis of three clinical trials revealed the benefit of SGLT2i in reducing the risk of cardiovascular death [33]. Other meta-analyses have also reported that SGLT2i reduced the risk of adverse kidney outcomes [34, 35]. These findings are consistent with those of our study, further demonstrating that SGLT2i are associated with lower risks of cardiovascular death and composite renal outcomes in patients with T2D who have received PAD revascularization. Regarding renal dysfunction in patients after PAD revascularization associated with higher risks of adverse cardiovascular, limb, and all-cause mortality events [12, 13], renal protection should be an important issue, that our data indicating the benefits of renal outcomes of SGLT2i. Our study indicated that SGLT2i were not associated with a lower risk of HFH compared with DPP4i, which is inconsistent with the results of previous landmark research [33]. A possible explanation for no reduction in HFH could be the fact that after propensity matching, on similar 6 to 7% in both cohorts had heart failure. Nevertheless, our previous study including a cohort of patients with T2D and concomitant PAD selected from the Taiwan NHIRD reported that compared with DPP4i, SGLT2i were associated with a lower risk of HFH [32]. Therefore, the inconsistency between the findings of the present study and those of the aforementioned landmark research may be due to differences in study design and settings or may be due to the fact that the benefits of SGLT2i over DPP4i in patients with advanced-stage PAD requiring revascularization are limited. Additional randomized and prospective studies are should be conducted to investigate the effects of SGLT2i on HFH in patients with T2D and advanced-stage PAD requiring revascularization.

This study has several limitations. First, although PSM was useful for balanced comparisons, it could not account for unknown confounders such as unmeasured variables, prescribing behavior, and medical adherence in this retrospective cohort study. In addition, the findings of observational studies should be interpreted with caution because they might be subject to time-related biases such as immortal time and time-lag biases, which may exaggerate the mortality effects. Accordingly, to avoid immortal time bias, we included only new prescriptions for SGLT2i or DPP4i after the date of PAD revascularization [36, 37]. Furthermore, to avoid time-lag bias from the prescriptions for the study drugs, we selected the same DPP4i agents as the comparator and included study patients with a similar disease stage such as that requiring PAD revascularization [29, 37]. Second, the NHIRD does not contain laboratory data such as glycohemoglobin (HbA1c) or serum creatinine levels. HbA1c levels have been reported to be associated with the risk of cardiovascular and limb events in patients with T2D [24–26, 38]. In addition, even after adjustment for CKD, the diagnosis of CKD based on coding could not represent the severity of CKD, which may influence the outcomes in patients with T2D [39]. Third, a low proportion of canagliflozin (6.23%) in the SGLT2i group had been studied in the cohort, that there might be insufficient evidence to support that canagliflozin does not increase the risk of amputation for patients with T2D who had undergone PAD revascularization. Fourth, miscoding and misclassification of underlying comorbidities and outcomes registered by each physician are another limitation of the study. Therefore, to improve the accuracy of study outcomes, we considered only primary discharge diagnoses in the cohort. Finally, we investigated only Asian patients, and whether our results can be extrapolated to other races remains unclear.

In conclusion, we observed that compared with DPP4i, SGLT2i were associated with lower risks of cardiac death and composite renal outcomes but were not associated with increased risks of adverse limb events in patients with T2D who had received PAD revascularization. Our study findings suggest that SGLT2i constitute an effective and safe alternative to DPP4i in such a high-risk population.

## Supplementary Information

**Additional file 1.** Additional figures and tables.

## Data Availability

The data that support the findings of this study are available from NHIRD but restrictions apply to the availability of these data, which were used under license for the current study and therefore are not publicly available. The SAS programs (codes) involved in this study are available from the corresponding authors upon reasonable request.
