# Supplementary material for: Adverse cardiovascular, limb, and renal outcomes in patients with diabetes after peripheral artery disease revascularization treated with sodium glucose cotransporter 2 inhibitors versus dipeptidyl peptidase-4 inhibitors
Source: Diabetol Metab Syndr. 2023 Jan 18;15:8. doi: 10.1186/s13098-023-00982-6 (PMC9847141; doi:10.1186/s13098-023-00982-6)
Supplement: Supplementary file 1 — Additional file 1. Additional figures and tables. [file 13098_2023_982_MOESM1_ESM.doc]

**SUPPLEMENTAL MATERIAL**

**Table S1**

***International Classification of Diseases (Ninth and Tenth Revisions) Clinical Modification* (*ICD-9-CM* and *ICD-10-CM*) codes used to define comorbidities and clinical outcomes in this** study

| **Disease** | ***ICD-9-CM* Codes** | ***ICD-10-CM* Codes** | **Diagnosis definition** |
| --- | --- | --- | --- |
| Ischemic stroke | 433, 434, 436 | I63, I64 | Discharge |
| Acute Myocardial infarction | 410 | I21-I23 | Discharge |
| Peripheral arterial disease | 440.0, 440.2, 440.3, 440.8, 440.9, 443, 444.0, 444.22, 444.8, 444.9, 447.9, 440.0, 38.08, 38.18, 38.38, 38.48, 38.68, 38.88, 39.50, 39.7, 39.90, 39.25, 39.26, 39.29, 84.10-84.15, 84.16-84.19 | I70.0, I70.2, I70.9, I70.3, I70.8, I75.89, I70.9, I73.0, I73.1, I73.8, I73.9, I79.1, I79.8, I74.01, I74.09, I74.3, I74.4, I74.5, I74.8, I74.9, I77.9, I70.0, 41, 045, 047, 049, 04B, 04C, 04H, 04J, 04L, 04N, 04P, 04Q, 04R, 04S, 04U, 04V, 04W  Location:(C,D,E,F,H,J,K,L,M,N,P,Q,R,S,T,U,V,W,Y)  0Y67, 0Y68, 0Y6C, 0Y6D, 0Y6F, 0Y6G, 0Y6H, 0Y6J, 0Y6M, 0Y6N, 0Y6P, 0Y6Q, 0Y6R, 0Y6S, 0Y6T, 0Y6U, 0Y6V, 0Y6W, 0Y6X, 0Y6Y | Discharge or Outpatient department ≥2 |
| Ischemic heart disease | 410, 411, 412, 413, 414 | I21-I25 | Outpatient department ≥2 |
| Congestive heart failure | 428 | I11.0, I13.0, I13.2, I42.0, I50, I50.1, I50.9 | Discharge |
| Hypertension | 401, 402 | I10-I16 | Outpatient department ≥2 |
| Diabetes mellitus | 250 | E11, E13 | Outpatient department ≥2 |
| Hyperlipidemia | 272 | E78 | Outpatient department ≥2 |
| Chronic gout | 274.0, 274.10, 274.11, 274.19, 274.81, 274.82, 274.89, 274.9 | M10, M1A | Outpatient department ≥2 |
| Chronic kidney disease | 580-589 | I12, I13, N00, N01, N02, N03, N04, N05, N07, N11, N14, N17, N18, N19, Q61 | Outpatient department ≥2 |
| Chronic liver disease | 570, 571, 572 | B150, B160, B162, B190, K704, K72, K766, I85 | Outpatient department ≥2 |
| Malignancy | 140.0-208.9 | C | Outpatient department ≥2 |
| Cardiac death | 390-392, 393-398, 410-414, 420-429 | I01, I02.0, I05-I09, I20-I25, I27, I30-I52 | the primary diagnosis during hospitalization or in emergency rooms |
| Composite renal outcomes:  (Dialysis/renal transplantation, death from renal causes, or hospitalization for renal events) | | | |
| Dialysis or renal transplantation |  | Z49, Z940, Z992 | Any position / inpatient admission or outpatient visit |
| Death from renal causes |  | E112, E132, E142, I120, I131, I132 N00-N08, N10-N16, N17, N18, N19, N20-N23, N25-N29 | Cause of death |
| Hospitalization for renal events |  | E112, E132, E142, I120, I131, I132, N17, N18, N19 | Primary diagnosis/inpatient admission |

Noted: *ICD-9-CM* codes were used during 2000–2015, and *ICD-10-CM* codes were used after 2016.

**Table S2*. International Classification of Diseases (9^th^ and 10^th^ edition) Clinical Modification* (*ICD-9-CM* and *ICD-10-CM*) codes used to define adverse limb outcomes in this study**

| Disease | *ICD-9-CM* Codes | *ICD-10-CM* Codes | Diagnosis definition |
| --- | --- | --- | --- |
| Revascularization  (Procedural codes) | 38.08 (incision of vessel, lower limb arteries) | 041, 045, 047, 049, 04B, 04C, 04H,  04J, 04L, 04N, 04P, 04Q, 04R, 04S,  04U, 04V, 04W  Location:(C, D, E, F, H, J, K, L, M, N, P, Q, R, S, T, U, V, W, Y) | Discharge |
|  | 38.18 (endarterectomy, lower limb arteries) |  |  |
|  | 38.38 (resection of vessel with anastomosis, lower limb arteries) |  |  |
|  | 38.48 (resection of vessel with replacement, lower limb arteries) |  |  |
|  | 38.68 (other excision of vessel, lower limb arteries) |  |  |
|  | 38.88 (other surgical occlusion of vessel, lower limb arteries) |  |  |
|  | 39.50 (angioplasty or atherectomy of noncoronary vessel) |  |  |
|  | 39.7 (Endovascular repair of vessel) |  |  |
|  | 39.90 (insertion of noncoronary artery stent) |  |  |
|  | 39.25 (aorta-iliac-femoral bypass) |  |  |
|  | 39.26 (other intra-abdominal vascular shunt or bypass) |  |  |
|  | 39.29 (other(peripheral) vascular shunt or bypass) |  |  |
| Amputation | - 1. (amputation of lower limb) | 0Y67, 0Y68, 0Y6C, 0Y6D, 0Y6F, 0Y6G, 0Y6H, 0Y6J, 0Y6M, 0Y6N, 0Y6P, 0Y6Q, 0Y6R, 0Y6S, 0Y6T, 0Y6U, 0Y6V, 0Y6W, 0Y6X, 0Y6Y |  |
|  | 84.10-84.15 low level amputation (amputation below knee) |  |  |
|  | 84.16-84.19 high level amputation (knee disarticulation or above) |  |  |

**Supplemental Figure Legend**

**Supplemental Figure I-VII**

**Subgroup analysis of the hazard ratios for the risks of ischemic stroke (Supplemental Figure I), acute myocardial infarction (Supplemental Figure II), heart failure hospitalization (Supplemental Figure III), cardiac death (Supplemental Figure IV), repeated revascularization (Supplemental Figure V), lower limb amputation (Supplemental Figure VI), and composite renal outcomes (Supplemental Figure VII) for SGLT2i versus DPP4i among patients with T2D who had undergone PAD revascularization after propensity score matching.**

Overall, the subgroup analysis revealed consistent results for most outcomes of SGLT2i versus DPP4i among patients aged ≥75 years, patients with CKD, female patients, and patients who used statins; these results were consistent with the main analysis results (*p* for interaction > 0.05; **Supplemental Figures I to VII**). The subgroup analysis indicated that SGLT2i reduced the risk of AMI, cardiac death, and composite renal outcomes in patients without concomitant metformin therapy but not in those with metformin therapy (*p* < 0.05; **Supplemental Figures II, IV, and VII**). The subgroup analysis of patients with concomitant CKD revealed a lower risk of repeated revascularization for SGLT2i versus DPP4i (*p* < 0.05; **Supplemental Figure V**).

Abbreviations: CKD = chronic kidney disease; DPP4i = dipeptidyl peptidase-4 inhibitors; SGLT2i = sodium-glucose co-transporter-2 inhibitors; T2D = type-2 diabetes

**Supplemental Figure I**

**
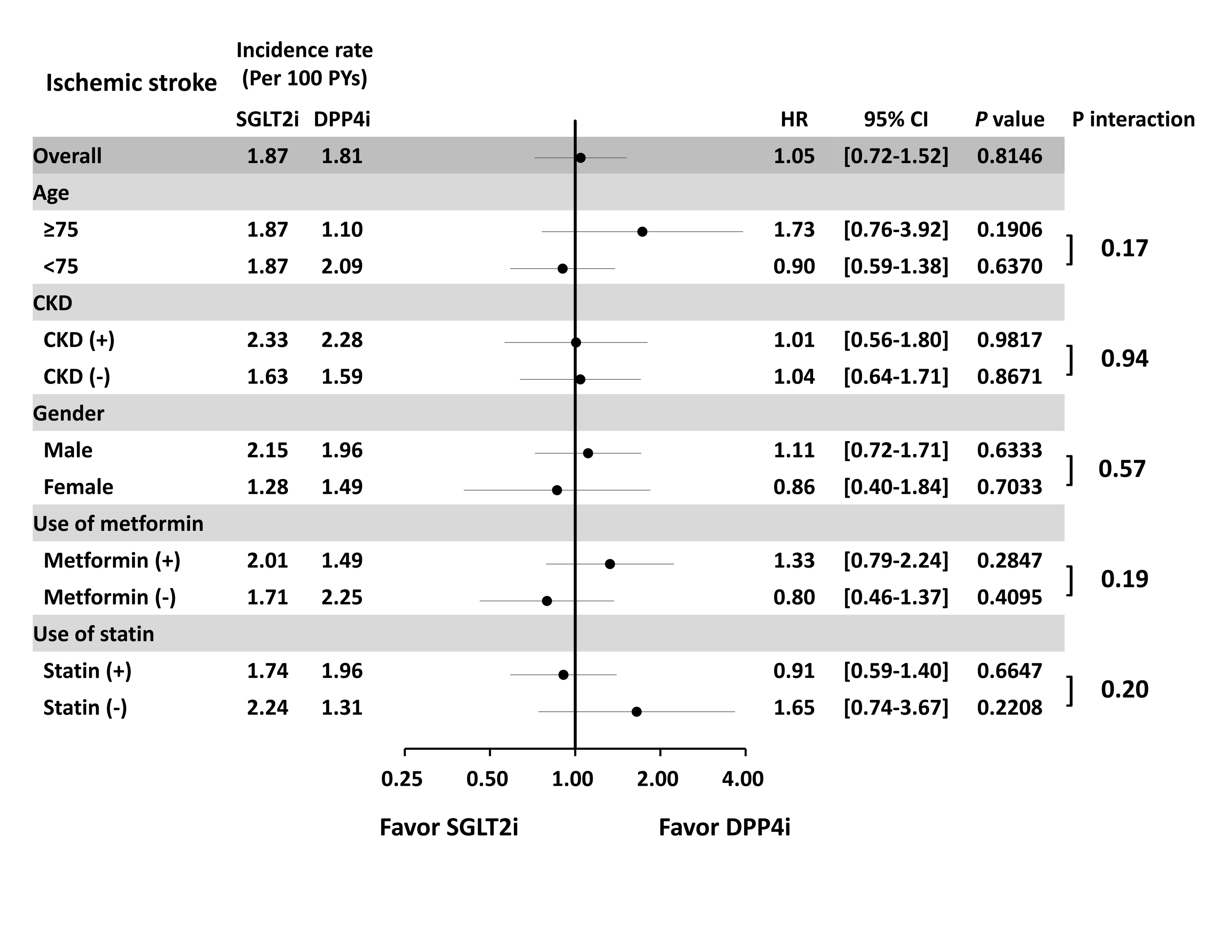
**

**Supplemental Figure II**


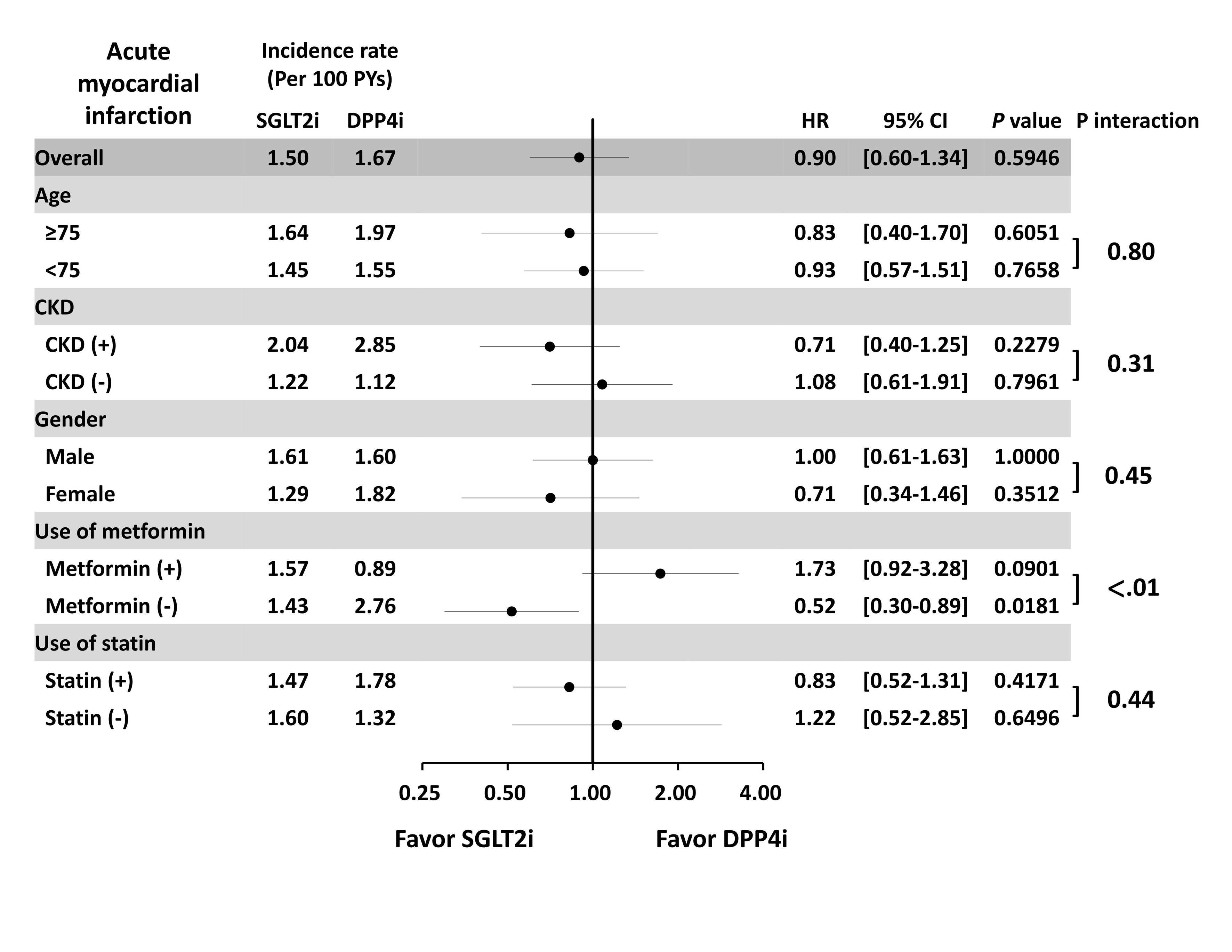


**Supplemental Figure III**

**
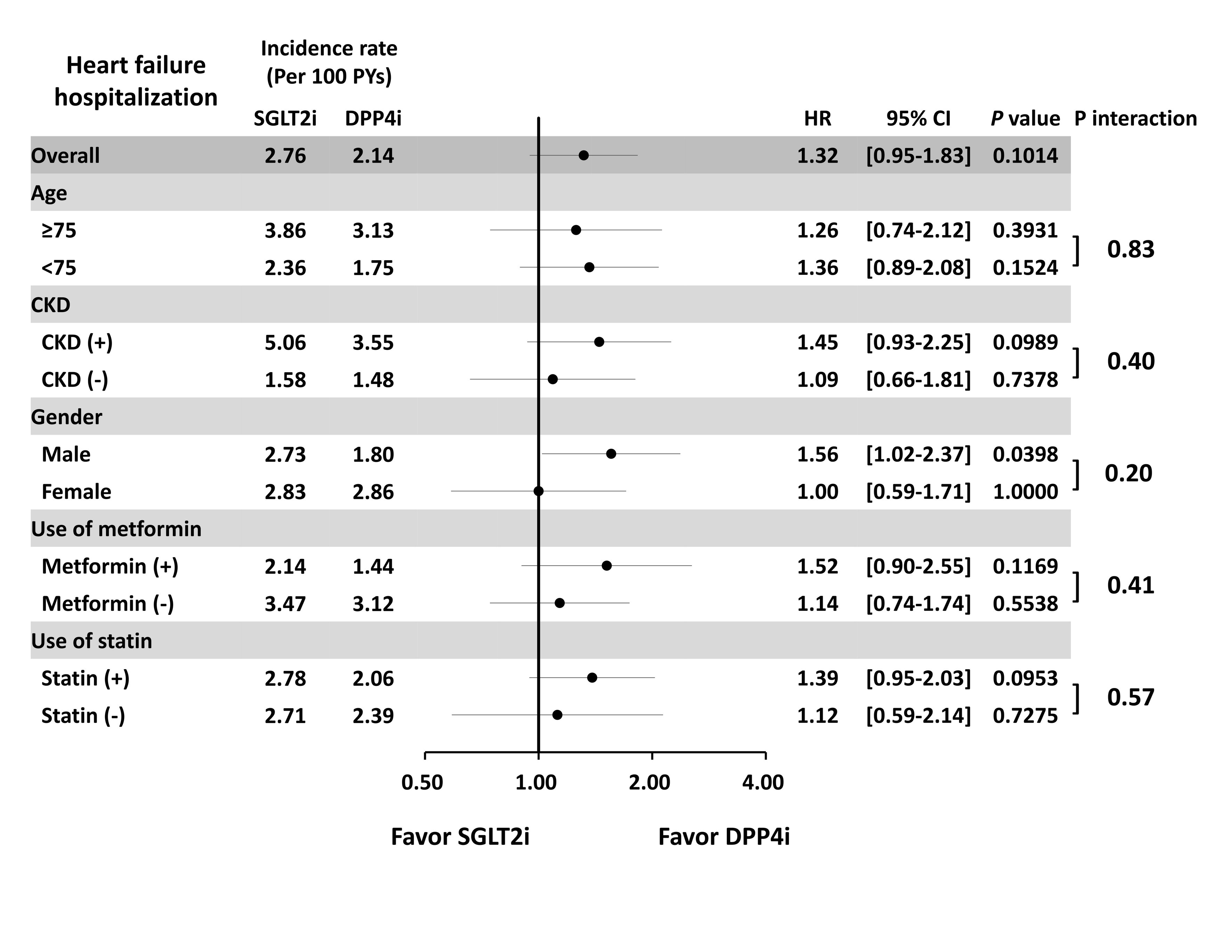
**

**Supplemental Figure IV**

**
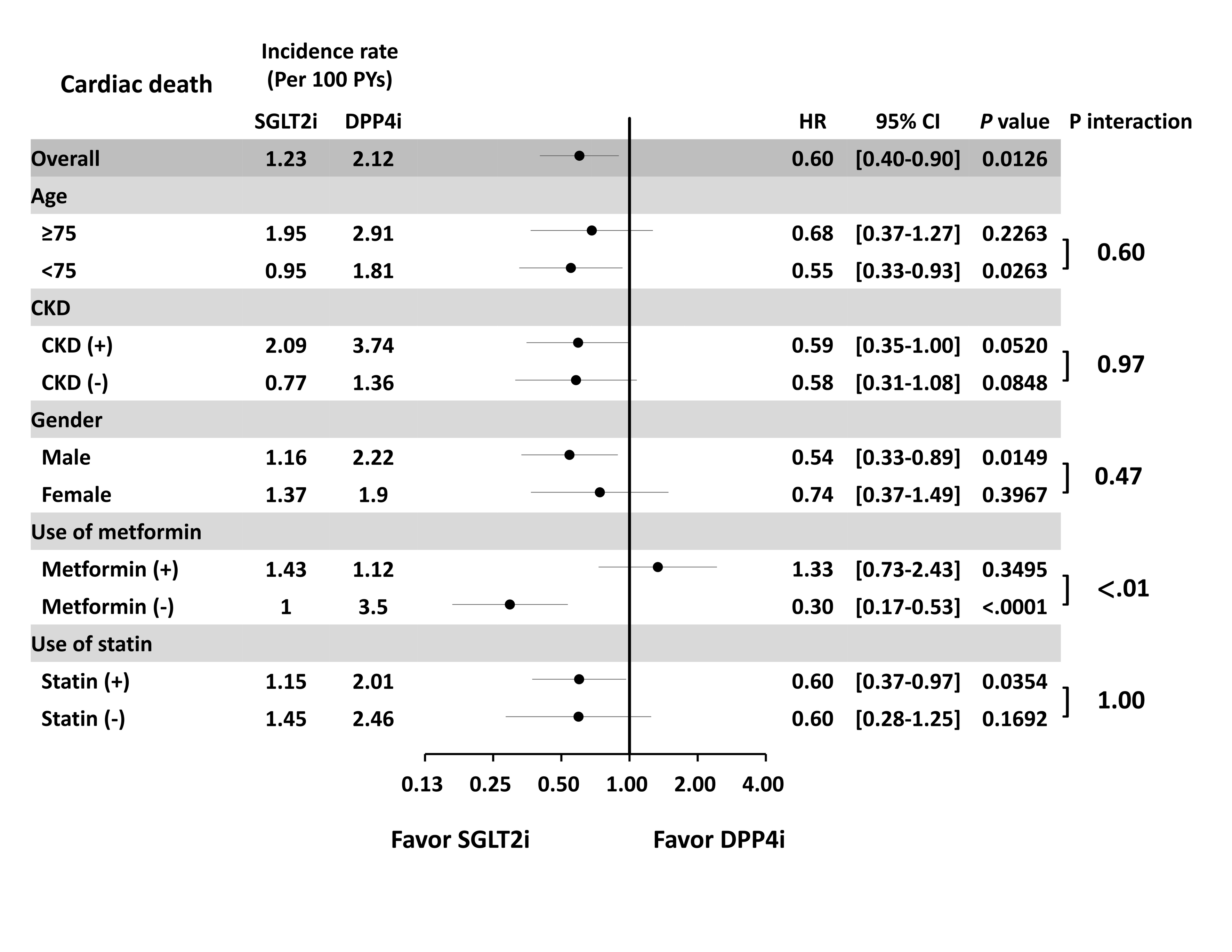
**

**Supplemental Figure V**

**
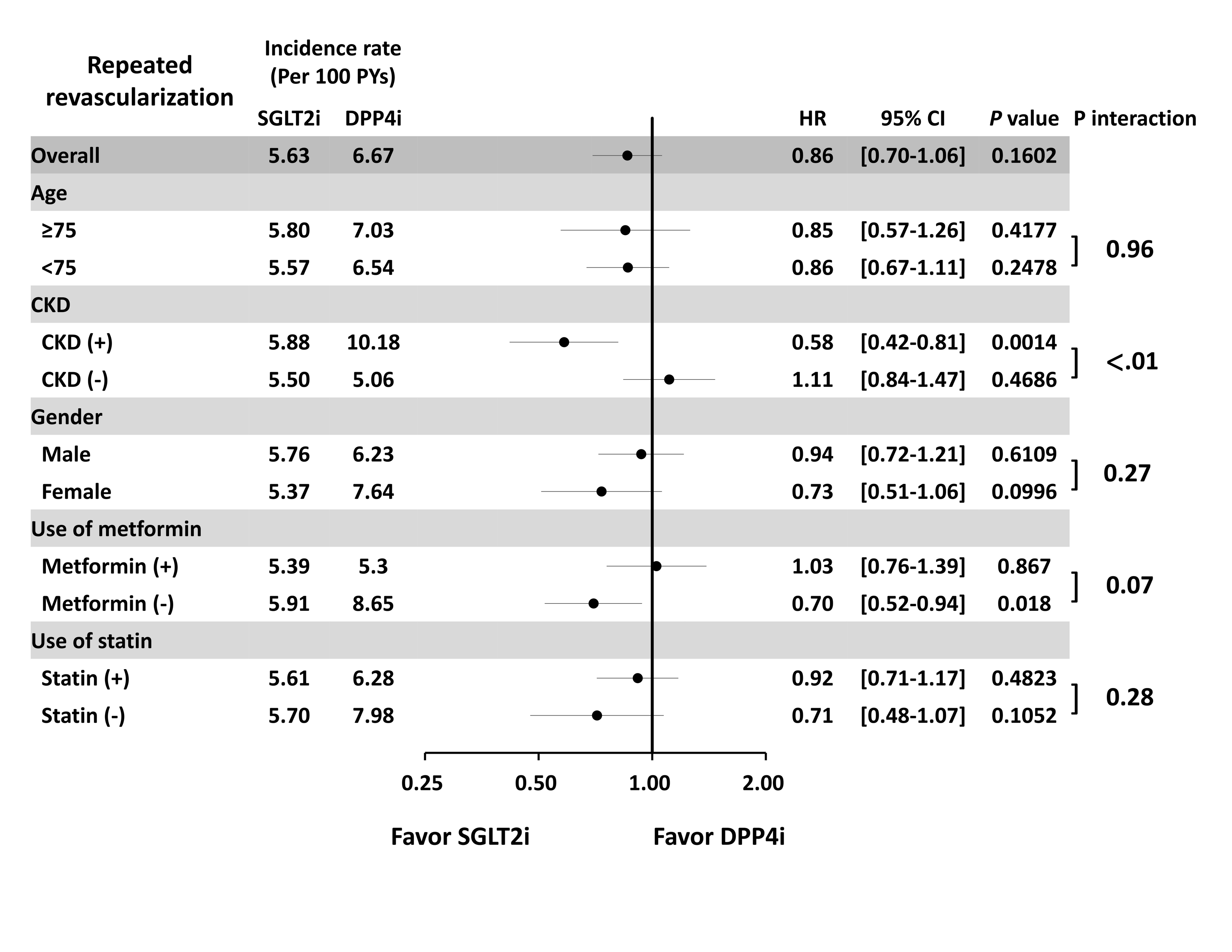
**

**Supplemental Figure VI**

**
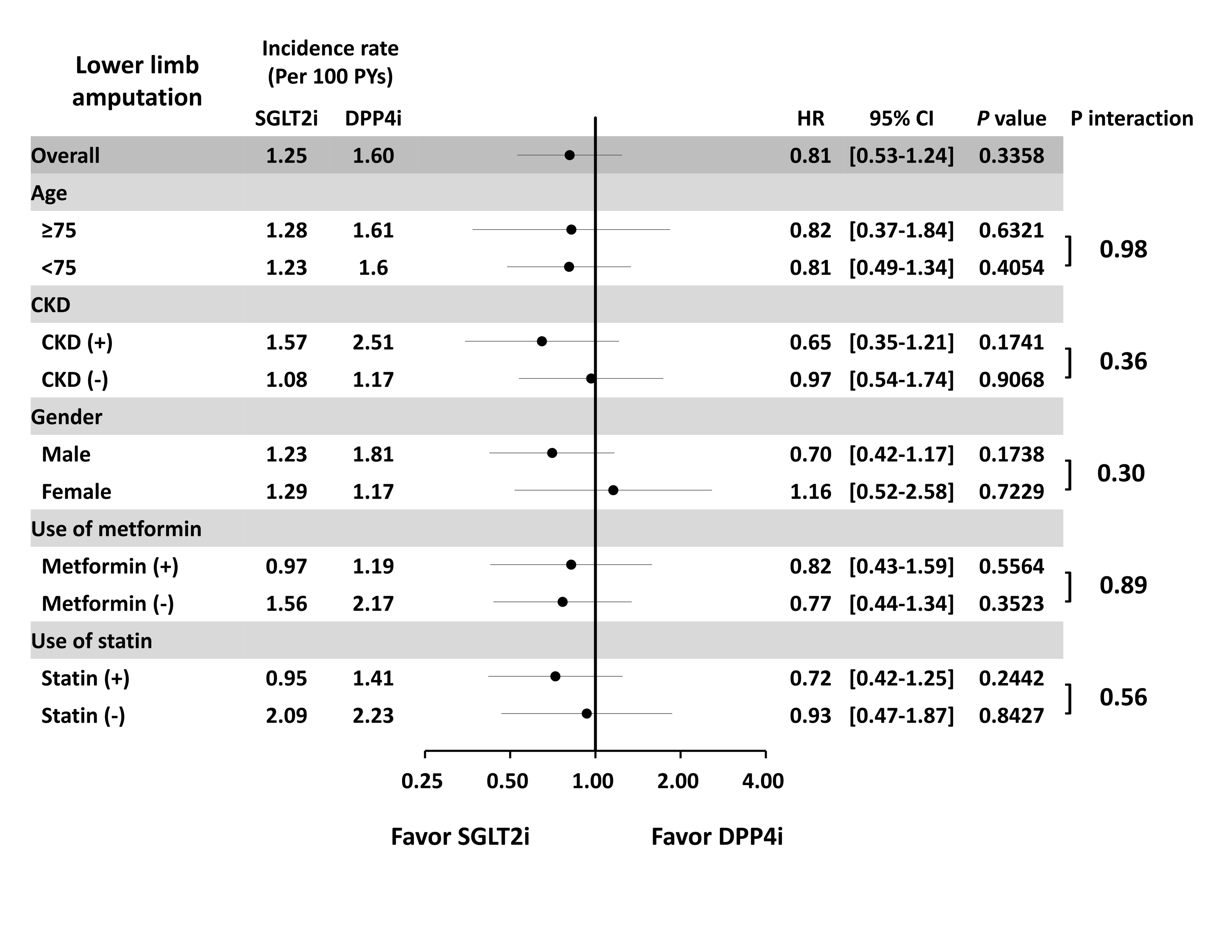
**

**Supplemental Figure VII**

**
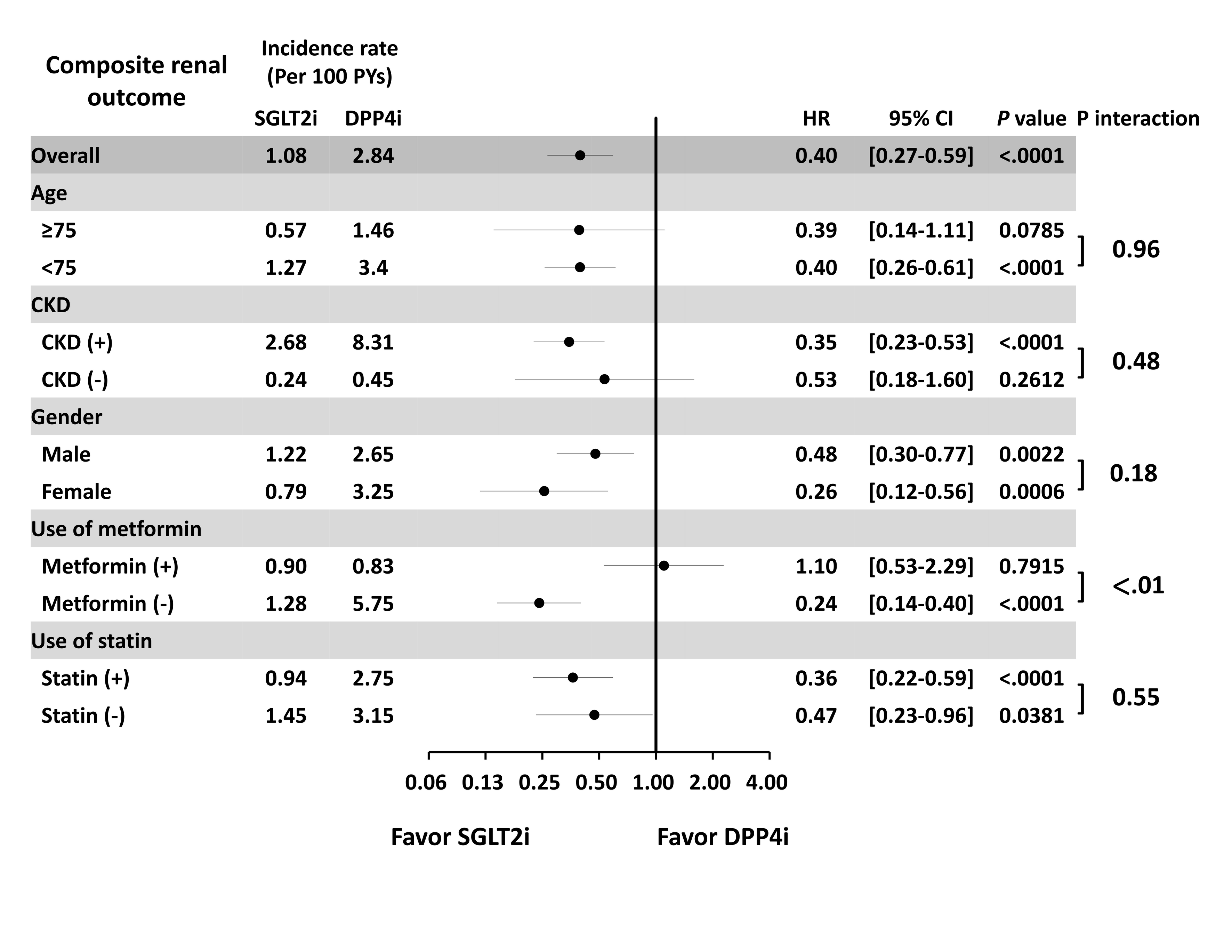
**
